# Clinical Effect of Iodine-125 Seed Implantation in Patients with Primary Liver Cancer and Its Effect on Th1/Th2 Cells in Peripheral Blood

**DOI:** 10.1155/2021/6199732

**Published:** 2021-07-29

**Authors:** Xiaoyan Chen, Fan Zhu, Bin Wang, Yu Zhou, Hao Xiong, Tao Fan, Wenjie Ke

**Affiliations:** ^1^Department of Hepatobiliary Surgery, Renmin Hospital of Wuhan University, Wuhan 430060, China; ^2^Department of Hepatobiliary Surgery, Ezhou Central Hospital, Ezhou 436099, China; ^3^Department of Outpatient, Chengdu Wuhou District Enxi Medical Cosmetology Hospital, Chengdu 610047, China; ^4^Department of Henia and Abdominal Wall Surgery, The Central Hospital of Wuhan, Tongji Medical College, Huazhong Univerity of Science and Technology, Wuhan 430030, China

## Abstract

**Objective:**

To investigate the clinical effect of iodine-125 seed implantation combined with chemotherapy in patients with primary liver cancer and the effect on peripheral blood Th1/Th2 cells.

**Methods:**

A total of 136 patients with primary liver cancer from April 2017 to June 2018 were selected as subjects and randomly divided into the control group and observation group with 68 cases in each group. The control group was treated with chemotherapy, and the observation group was treated with iodine-125 seed implantation on the basis of the control group. After 3 months of treatment, the curative effect was investigated. Serum tumor markers, peripheral blood Th1/Th2 cells, and side effects and recurrence rate were compared between the two groups.

**Results:**

The levels of serum tumor markers in both groups at 3 months after treatment were lower than before treatment (*P* < 0.05). Three months after treatment, the levels of tumor markers AFP, AFP-L3, and GP73 in the observation group were 14.61 ± 3.49 *μ*g/L, 3.29 ± 0.41 ng/mL, and 51.24 ± 4.51 *μ*g/L, respectively, which were lower than those in the control group, 32.53 ± 4.59 *μ*g/L, 5.63 ± 0.63 ng/mL, and 71.52 ± 6.05 *μ*g/L (*P* < 0.05). At 3 months after treatment, the level of including interleukin-2 (IL-2) and tumor necrosis factor-*α* (TNF-*α*) in Th1 cells of the observation group was higher than that of the control group (*P* < 0.05), whereas the levels of IL-4, IL-6, and IL-10 in Th2 cells were lower than those in the control group (*P* < 0.05). There was no statistical significance in the incidence of leukopenia, thrombocytopenia, and gastrointestinal reactions between the two groups (*P* > 0.05). The recurrence rate of the observation group at 12, 24, and 36 months after treatment was lower than that of the control group (*P* < 0.05).

**Conclusion:**

Iodine-125 seed implantation combined with chemotherapy in patients with primary liver cancer can reduce the level of serum tumor markers, improve the level of peripheral blood Th1/Th2 cells, and reduce the recurrence rate of patients without increasing the incidence of side effects, which is worthy of promoting the application of iodine-125 seed implantation.

## 1. Introduction

Primary liver cancer is a common malignant tumor in clinical practice, which tends to occur in people aged 40–50 years, and the incidence of in primary liver cancer in men is slightly higher than that in women. The incidence of primary liver cancer was slightly higher in men than in women. The pathogenesis of primary liver cancer has not been clarified, but is generally believed to be related to cirrhosis viral hepatitis and chemical carcinogens such as aflatoxin [1]. Due to the lack of typical clinical symptoms in the early stage of primary liver cancer, with the prolongation of the course of the disease, the clinical manifestations are mostly liver pain, systemic and digestive tract symptoms, and liver enlargement, leading to great difficulty in clinical diagnosis and treatment; therefore, most patients are diagnosed in the middle and late stages [2, 3]. Chemotherapy is a common method for the clinical treatment of primary liver cancer. Chemotherapy drugs can kill tumor cells and prolong the life of patients [4]. However, the incidence of side effects of chemotherapy is high, and drugs can affect normal cells, leading to poor treatment tolerance and compliance of patients [5, 6]. Iodine-125 (^125^I) seed implantation can continuously emit gamma rays through implanting ^125^I seeds from a tiny radioactive source, which can damage tumor cells, cause DNA double-strand emission break, and cause the loss of the proliferation ability of tumor cells [7, 8].

However, there are few clinical studies on the effect of iodine-125 seed implantation combined with chemotherapy on the level of Th1/Th2 cells in peripheral blood of patients with primary liver cancer. Therefore, this study took patients with primary liver cancer as the object to explore the clinical effect of iodine-125 seed implantation combined with chemotherapy in patients with primary liver cancer.

## 2. Materials and Methods

### 2.1. Clinical Data

A total of 136 patients with primary liver cancer from April 2017 to June 2018 at Renmin Hospital of Wuhan University, Wuhan, China, were prospectively selected as the subjects and randomly divided into two groups, control group and observation group. In the control group, there were 68 patients, 41 males and 27 females, age 44–68 years, average 56.71 ± 5.69 years old. Body mass index (BMI) was 18–29 kg/m^2^, with an average of 23.51 ± 3.48 kg/m^2^. Liver function grade: grade A (13 cases), grade B (32 cases), and grade C (23 cases). Complications: 4 cases of hypertension, 6 cases of diabetes, and 5 cases of hyperlipidemia. In the observation group, there were 68 patients, 39 males and 29 females, aged 43–69 years, with an average of 56.79 ± 5.83 years old. BMI was 17–28 kg/m^2^, with an average of 23.58 ± 3.52 kg/m^2^. The liver function grade: grade A (11 cases), grade B (33 cases), and grade C (24 cases). Complications: 5 cases of hypertension, 4 cases of diabetes, and 6 cases of hyperlipidemia. The demographic characteristics of patients are shown in Table 1. This study was approved by the ethics committee of the Renmin Hospital of Wuhan University, and all patients signed informed consent.

### 2.2. Inclusion and Exclusion Criteria

The inclusion criteria were as follows: (1) all patients met the diagnostic criteria for primary liver cancer [9, 10] and were diagnosed by CT-guided percutaneous liver biopsy. (2) It meets the indications for iodine-125 seed implantation combined with chemotherapy and is tolerated by all patients. (3) Complete baseline and follow-up data were available. The exclusion criteria were as follows: (1) patients with mental abnormalities and other malignant tumors or associated with autoimmune system diseases; (2) patients with severe cardiac and renal dysfunction or abnormal coagulation function; (3) metastatic hepatic carcinoma or liver cyst.

### 2.3. Methods

After admission, both groups were routinely given liver-protective treatment to strengthen nutritional support intervention for patients. Liver-protective drugs were routinely given to patients with cirrhosis to improve liver function and inhibit the progression of cirrhosis. At the same time, routine antiviral treatment intervention was strengthened for patients [11]. The right femoral artery was intubated through the skin. After a successful puncture, the catheter was selectively intubated and reached the common hepatic artery. Digital subtraction angiography (DSA) was completed, intrahepatic tumor staining was visible, and chemotherapy drugs were injected. Chemotherapy drugs used were epirubicin (Shenzhen Main Luck Pharmaceuticals Inc., National Drug Approval no. H10930106, specification: 20 mg) 40–60 mg, 5-fluorouracil (Heilongjiang Fuhe Huaxing Pharmaceutical Group Co., Ltd., National Drug Approval no. H23021711, specifications:10 mL, 0.25 g) 0.75–1 g, mitomycin (Jiangsu Hengrui Medicine Co., Ltd., National Drug Approval no. H20023070) 6–12 mg, and 30 mL superliquefied iodide oil (Guerbet, Hong Kong, China). The patients were treated once every 4 weeks for 3 months (3 courses) [12].

Observation group: on the basis of the control group, iodine-125 seed implantation was combined. The radioactive seed implantation planning system was used to delineate the treatment target area (i.e., primary tumor and intrahepatic metastases) and set the puncture path for ^125^I seed, and the treatment was carried out according to the principle of 100.0% prescription dose covering 95.0% planned target area to determine the quantity and dose of iodine-125 seed implantation. After the above procedures, ^125^I seeds were routinely implanted into the lesions with the aid of a particle gun according to the implantation plan, followed by CT scan for reexamination and verification. After 3 months of treatment, the curative effect was investigated. All patients were followed up for 36 months.

### 2.4. Observation Indicators

1. Serum tumor markers: peripheral fasting blood (3 mL) was collected on the day before treatment and the day after 3 months of treatment, respectively. Peripheral fasting blood was stored at −80°C. After centrifugation, the electrochemiluminescence was carried out to determine the level of alpha-fetoprotein (AFP) [13]. Enzyme-linked immunosorbent assay was utilized to determine the levels of alpha-fetoprotein variants (AFP-L3) and Golgi body protein 73 (GP73) [14]. (2) Th1/Th2 cells in peripheral blood: serum specimens were collected from the isolated peripheral blood samples and then stored at −80°C. The levels of Th1 (including interleukin-2 (IL-2) and tumor necrosis factor-*α* (TNF-*α*)) and Th2 cells (interleukin-4, 6, and 10) in peripheral blood of patients were determined by the enzyme-linked immunosorbent assay [15, 16]. (3) Side effect and recurrence rates: the incidence of leukopenia, thrombocytopenia, and gastrointestinal reactions in the two groups was recorded. After the treatment, both groups were followed up for 36 months, and the recurrence rates were recorded at 12, 24, and 36 months after the treatment.

### 2.5. Statistical Analysis

SPSS 24.0 software was applied to analyze the data. The enumeration data were tested by *χ*^2^ test and represented by *N* (%), while the measurement data were tested by *t*-test and represented by ($\bar{x}\pm s$). *P* < 0.05 was considered statistically significant.

## 3. Results

### 3.1. Comparison of Tumor Markers before and after Treatment

Before treatment, the levels of tumor markers in the two groups were not statistically significant (*P* > 0.05). Three months after treatment, the levels of serum tumor markers in both groups were lower than those before treatment (*P* < 0.05). Three months after treatment, the levels of tumor markers (AFP, AFP-L3, and GP73) in the observation group were lower than those in the control group (*P* < 0.05), as shown in Table 2.

### 3.2. Comparison of Th1/Th2 Cells between the Two Groups

Before treatment, the levels of Th1/Th2 cells were not statistically significant between the observation group and control group (*P* > 0.05). Three months after treatment, the level of Th1 cells in both groups was higher than that before treatment (*P* < 0.05). The level of Th2 cells was lower than before treatment (*P* < 0.05). The level of IL-2 and TNF-*α* in Th1 cells of the observation group was higher than that of the control group at 3 months after treatment (*P* < 0.05), whereas the levels of IL-4, IL-6, and IL-10 in Th2 cells of the observation group were lower than those in the control group (*P* < 0.05), as shown in Table 3.

### 3.3. Comparison of Side Effects and Recurrence Rate between the Two Groups

There was no statistical significance in the incidence of leucopenia, thrombocytopenia, and gastrointestinal reactions between the observation group and control group (*P* > 0.05). The recurrence rate of the observation group at 12, 24, and 36 months after treatment was lower than that of the control group (*P* < 0.05), as shown in Table 4.

## 4. Discussion

Chemotherapy is the preferred treatment for patients with primary liver cancer; transarterial chemoembolization (TACE) is the main treatment [17, 18]. High concentration of chemotherapeutic drugs can be injected into the tumor lesions, and then the tumor supplying blood vessels can be embolized through an embolization agent, which can make the tumor inactivated under the dual effects of ischemia and hypoxia. At the same time, through the injection of chemotherapy drugs, the effect of toxic inactivating of tumor cells can be achieved, playing a good antitumor effect. Previous studies showed that the application of TACE in patients with primary liver cancer is helpful to improve the quality of life, but with the extension of the chemotherapy cycle, the incidence of clinical drug resistance is higher, leading to poor patient prognosis [19]. In recent years, iodine-125 seed implantation combined with chemotherapy has been applied in patients with primary liver cancer with satisfactory results [20, 21]. In this study, 3 months after treatment, the levels of tumor markers (AFP, AFP-L3, and GP73) in the observation group were lower than those in the control group (*P* < 0.05), indicating that iodine-125 seed implantation combined with chemotherapy is beneficial to reduce the level of tumor markers in patients with primary liver cancer and is conducive to the recovery of patients. Iodine-125 seed implantation continuously emits gamma rays, which is conducive to the destruction of tumor tissue, causes the DNA double-strand emission break, and causes the loss of tumor cell activity [22–24]. At the same time, *γ*-rays have a direct damage effect on the tumor DNA molecular chain, can also ionize water molecules in the body, produce free radicals and biological molecular interaction, and further aggravate the tumor tissue cell damage. Some Chinese scholars [25] took 76 patients with advanced primary liver cancer as the subjects and gave them chemotherapy combined with iodine-125 seed implantation. The results showed that the tumor control rate of patients was 85.8%. In this study, the incidence of leucopenia, thrombocytopenia, and gastrointestinal reactions between the observation group and control group had no statistical significance, whereas the recurrence rate of the observation group at 12, 24, and 36 months after treatment was lower than that of the control group (*P* < 0.05), indicating that iodine-125 seed implantation combined with chemotherapy in the treatment of primary liver cancer is safe and can improve the survival of patients.

Helper T lymphocytes play a crucial role in the occurrence and development of primary liver cancer. Clinically, helper T lymphocytes can be divided into two subsets, Th1 and Th2, according to the cytokines secreted by helper T lymphocytes. Th1 can secrete IL-2 and TNF-*α*, whereas Th2 cells can secrete IL-4, IL-6, and IL-10. In the normal human body, Th1 and Th2 maintain a dynamic balance [26]. Previous studies [27] have shown that Th1 can enhance the cytotoxic effect of killing cells and mediate cellular immunity. On the contrary, Th2 can promote the production of antibodies, inhibit Th1 cells, and mediate humoral immunity. For patients with primary liver cancer, Th1 cells will be in a dominant state, and the body has active immunity to the tumor. However, with the continuous development of the disease course, Th1 cells will drift to Th2 cells, and the antitumor response of the body will be inhibited. Therefore, the current study investigated the effect of iodine-125 seed implantation combined with chemotherapy on the level of Th1/Th2 in peripheral blood of patients with primary liver cancer. The results showed that the level of IL-2 and TNF-*α* in Th1 cells in the observation group was higher than that in the control group at 3 months after treatment (*P* < 0.05), whereas the levels of IL-4, IL-6, and IL-10 in Th2 cells were lower than those in the control group (*P* < 0.05) indicating that iodine-125 seed implantation combined with chemotherapy in patients with primary liver cancer can maintain the balance of Th1/Th2 cells in peripheral blood, enhance the body's immunity, and facilitate the recovery of patients. Clinically, iodine-125 seed implantation combined with chemotherapy in patients with primary liver cancer can exert the advantages of different treatment methods, help to maintain the body's immune level, and achieve good results. In this study, the recurrence rate of the observation group at 12, 24, and 36 months after treatment was lower than that of the control group (*P* < 0.05).

We are the first to study the changes of Th1/Th2 cell levels in peripheral blood of patients with iodine-125 seed implantation combined with chemotherapy in the treatment of primary liver cancer and, for the first time, to elucidate the effect of this combination therapy on the immune system. However, the mechanism of its influence still needs further study.

In conclusion, iodine-125 seed implantation combined with chemotherapy in patients with primary liver cancer can reduce the level of serum tumor markers, improve the level of Th1/Th2 cells in peripheral blood, and reduce the recurrence rate of patients without increasing the incidence of side effects, which is worthy of promotion and application.

## Figures and Tables

**Table 1:** The demographic characteristics of patients.

Demographic characteristics (*n*)	Control group (*n* = 68)	Observation group (*n* = 68)
Male	41	39
Female	27	29
Age ($\bar{\chi }$ ± SD)	44–68 (56.71 ± 5.69)	43–69 (56.79 ± 5.83)
BMI ($\bar{\chi }$ ± SD)	18–29 (23.51 ± 3.48)	17–28 (23.58 ± 3.52)

Liver function grade		
Grade A	13	11
Grade B	32	33
Grade C	23	24

Complications		
Hypertension	4	5
Diabetes	6	4
Hyperlipidemia	5	6

**Table 2:** Comparison of tumor markers before and after treatment.

Group	*n*	AFP (ug/L)		AFP-L3 (ng/mL)		GP73 (ng/mL)	
		Before treatment	Three months after treatment	Before treatment	Three months after treatment	Before treatment	Three months after treatment
Observation group	68	57.93 ± 5.61	14.61 ± 3.49^#^	8.12 ± 0.69	3.29 ± 0.41^#^	92.41 ± 7.46	51.24 ± 4.51^#^
Control group	68	57.97 ± 5.64	32.53 ± 4.59^#^	8.14 ± 0.72	5.63 ± 0.63^#^	92.45 ± 7.49	71.52 ± 6.05^#^
*T*	—	1.593	7.846	0.951	6.315	1.229	8.893
*P*	—	0.315	0.000	0.692	0.000	0.646	0.000

^#^ *P* < 0.05 vs. before treatment.

**Table 3:** Comparison of Th1/Th2 cell levels between the two groups.

Group		IL-2 (pg/mL)	TNF-*α* (pg/mL)	IL-4 (pg/mL)	IL-6 (pg/mL)	IL-10 (pg/mL)
Observation group (*n* = 68)	Before treatment	16.92 ± 2.51	1.28 ± 0.51	174.34 ± 11.69	19.43 ± 3.21	9.83 ± 0.52
	Three months after treatment	23.59 ± 3.69^#*∗*^	1.74 ± 0.64^#*∗*^	98.87 ± 15.41^#*∗*^	13.23 ± 2.14^#*∗*^	4.26 ± 0.43^#*∗*^

Control group (*n* = 68)	Before treatment	16.94 ± 2.54	1.29 ± 0.53	176.41 ± 24.81	19.45 ± 3.24	9.84 ± 0.54
	Three months after treatment	20.12 ± 3.24^*∗*^	1.45 ± 0.59^*∗*^	123.59 ± 18.42^*∗*^	16.69 ± 3.02^*∗*^	7.19 ± 0.49^*∗*^

^#^ *P* < 0.05 vs. the control group. ^*∗*^*P* < 0.05 vs. before treatment.

**Table 4:** Comparison of side effects and recurrence rate between the two groups.

Group	*n*	Side effect			Recurrence rate		
		Leucopenia	Thrombocytopenia	Gastrointestinal reactions	12 months	24 months	36 months
Observation group	68	3 (4.41)	4 (5.88)	2 (2.94)	1 (1.47)	4 (5.88)	8 (11.76)
Control group	68	4 (5.88)	5 (7.35)	1 (1.47)	7 (10.29)	17 (25.00)	24 (35.29)
*x* ^2^	—		0.061		4.781	9.517	10.462
*P*	—		0.805		0.029	0.002	0.001

## Data Availability

The data used to support the findings of this study are available from the corresponding author upon request.
